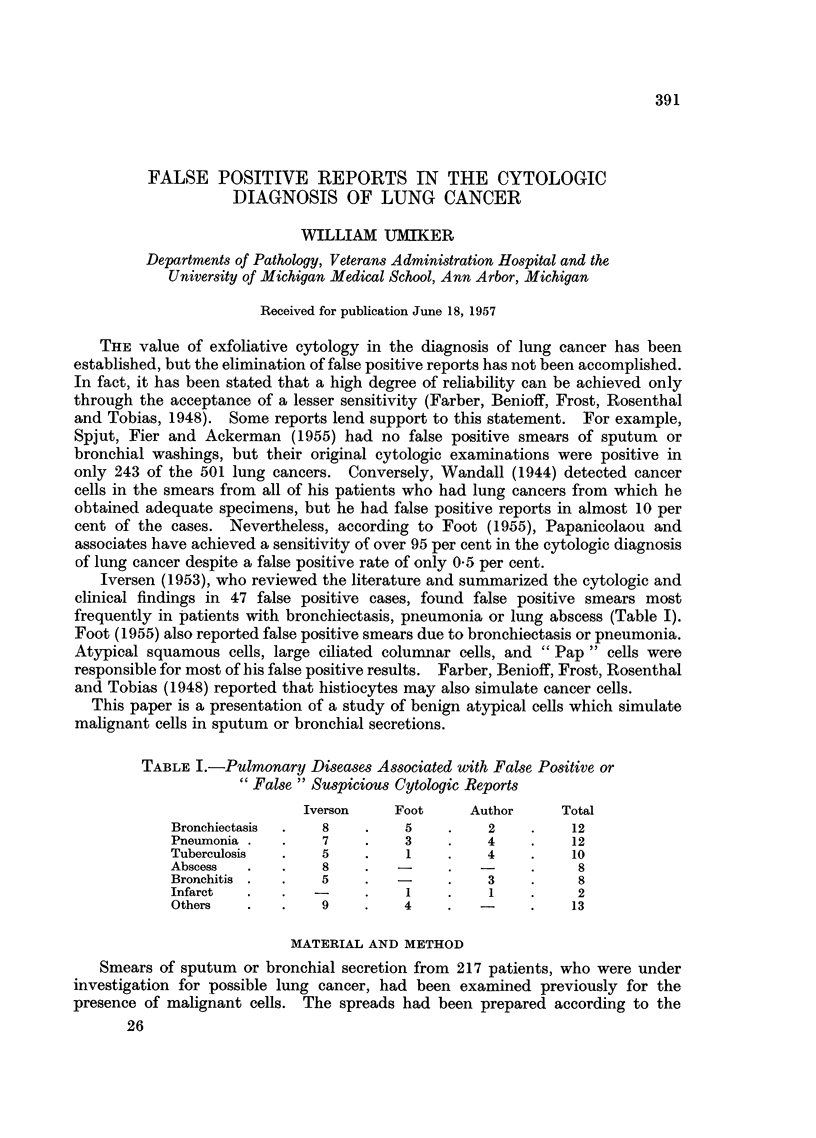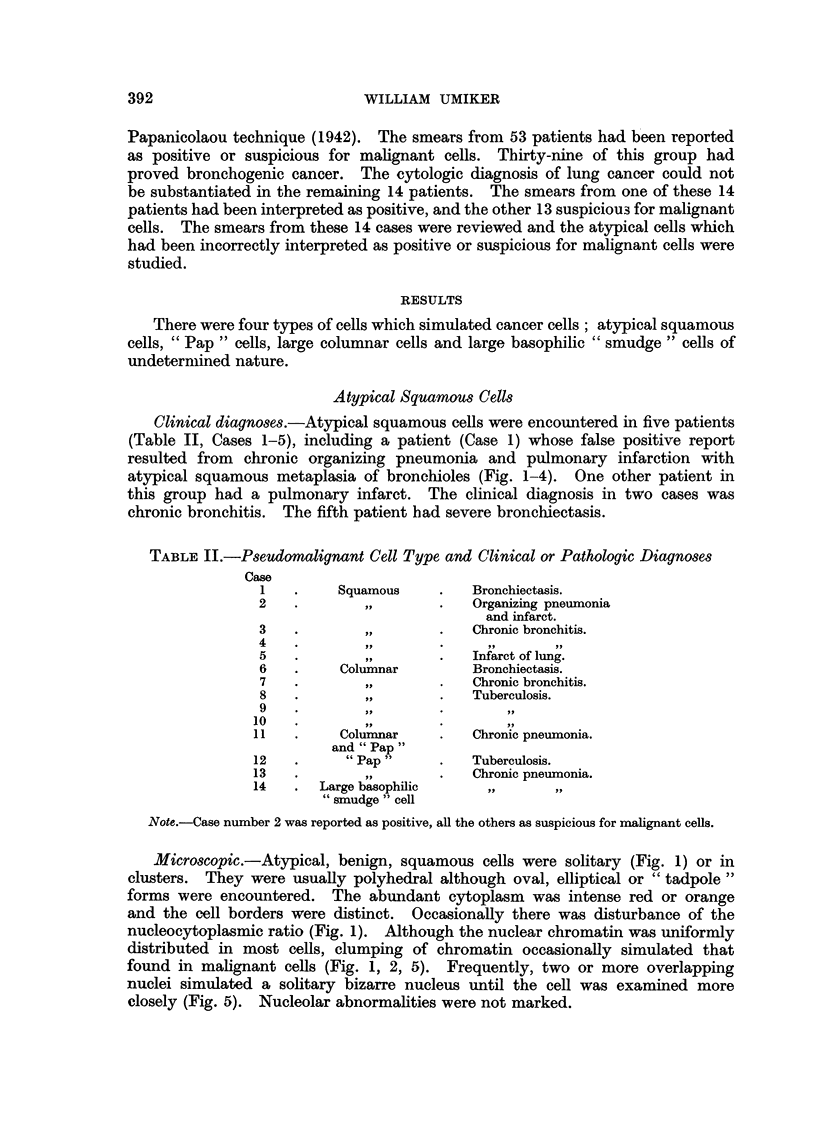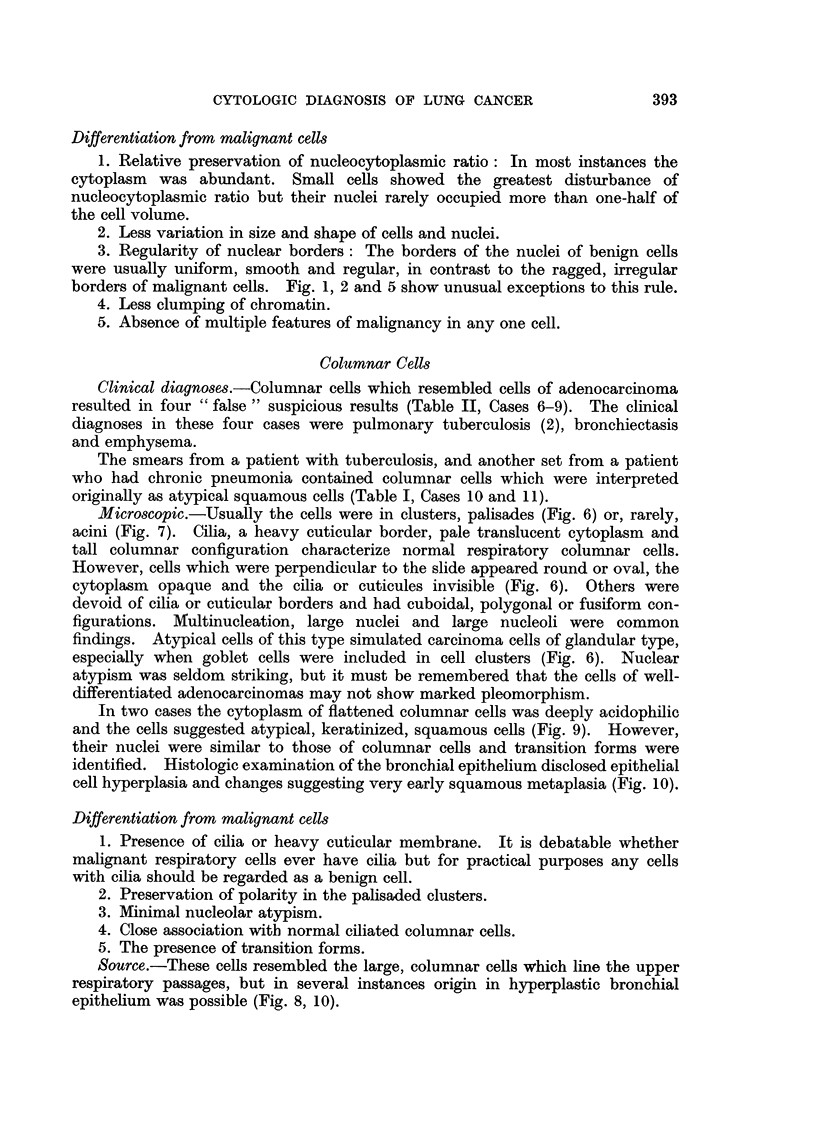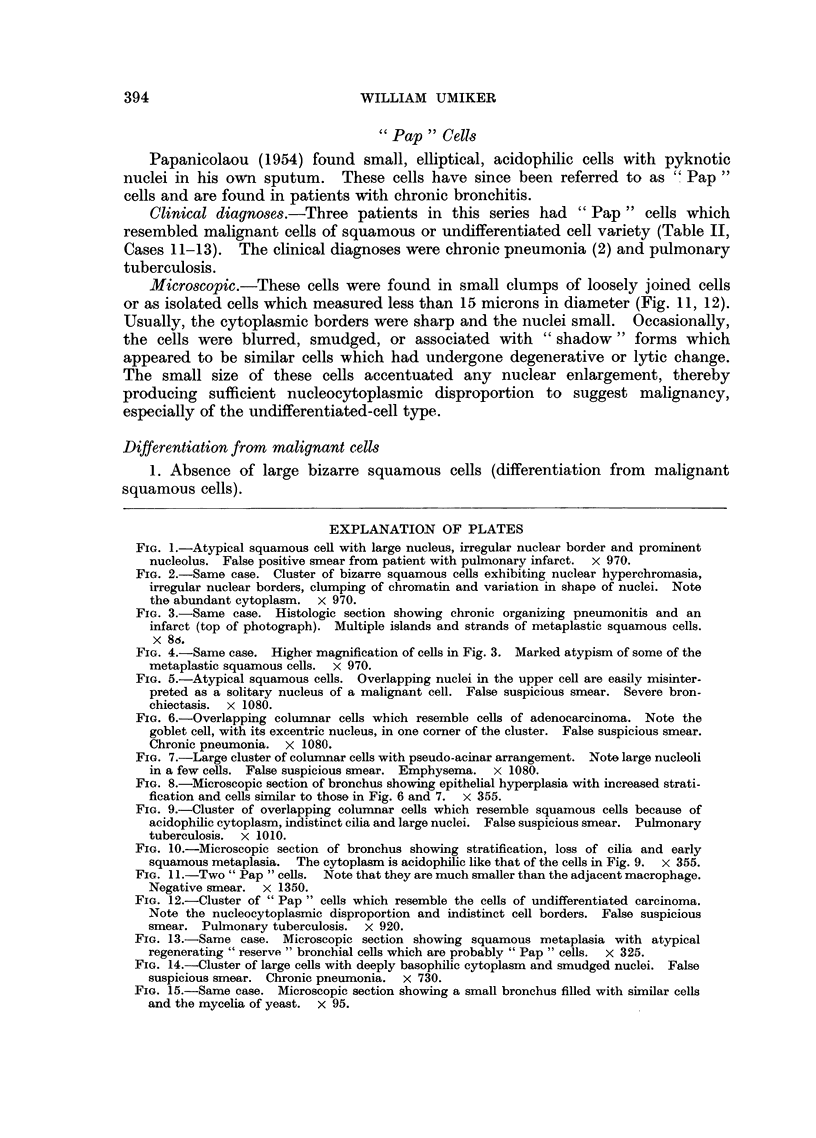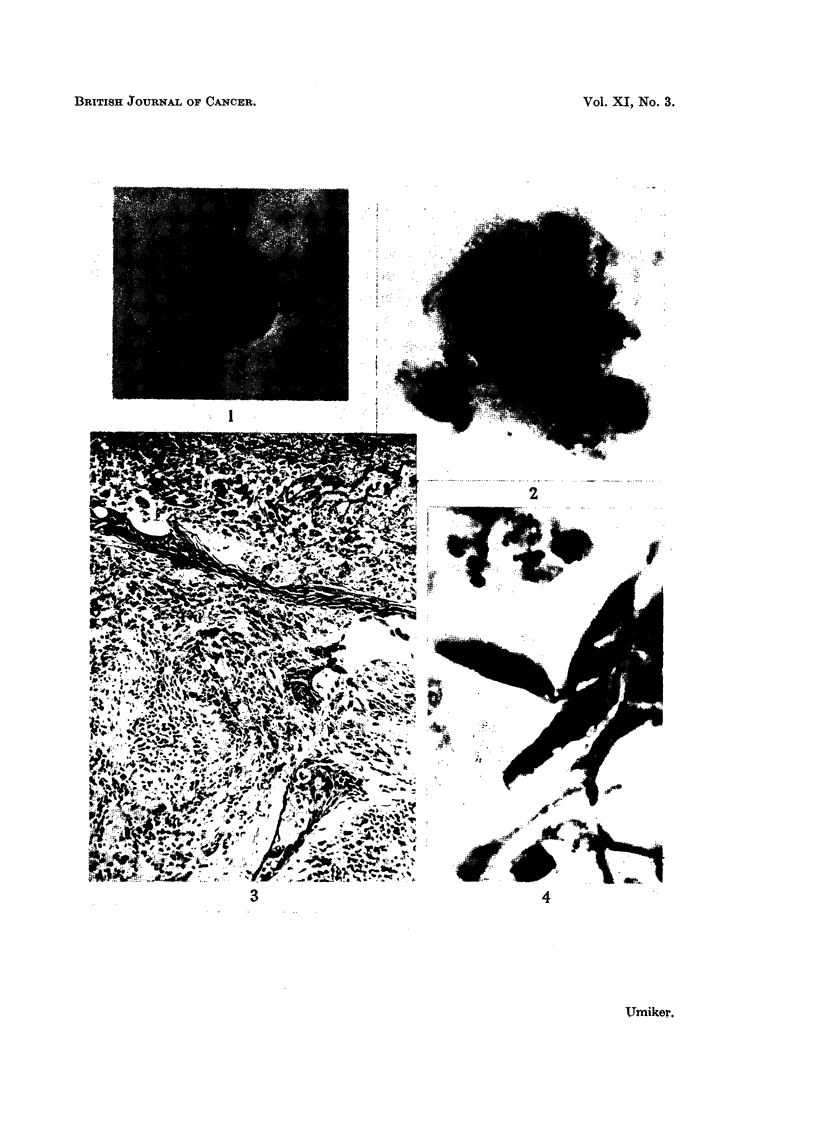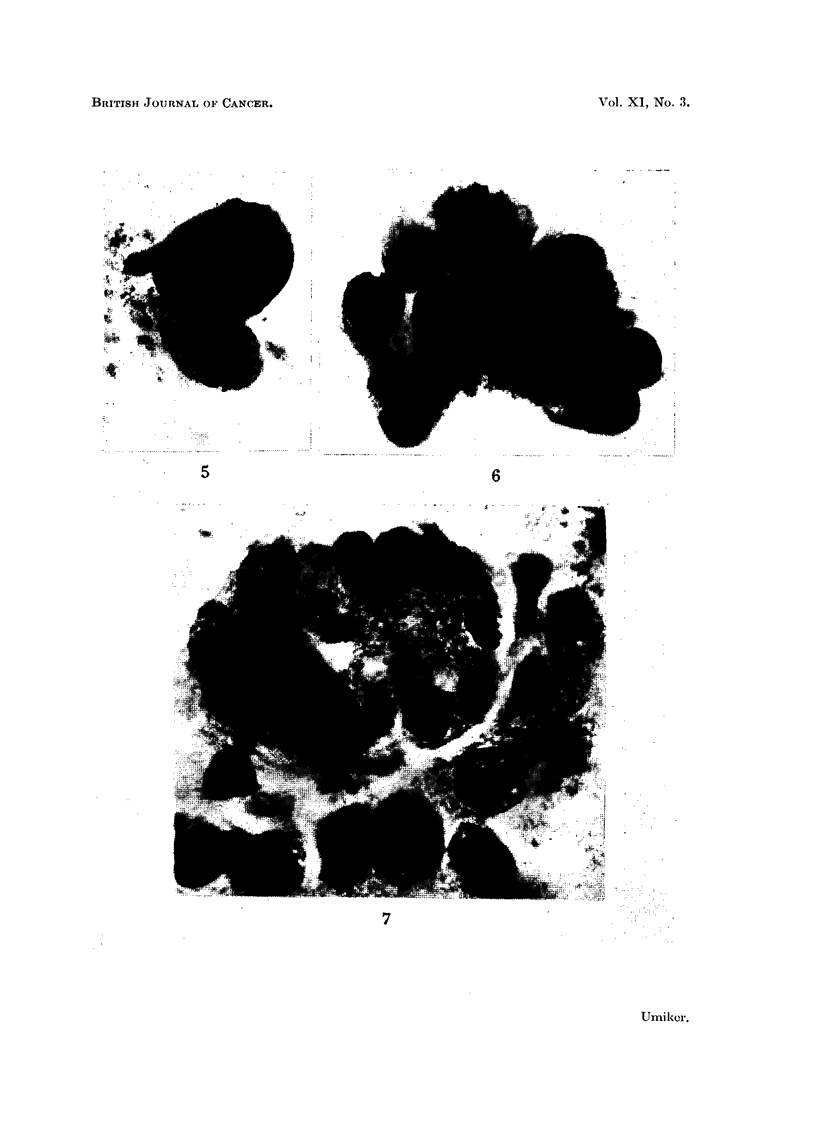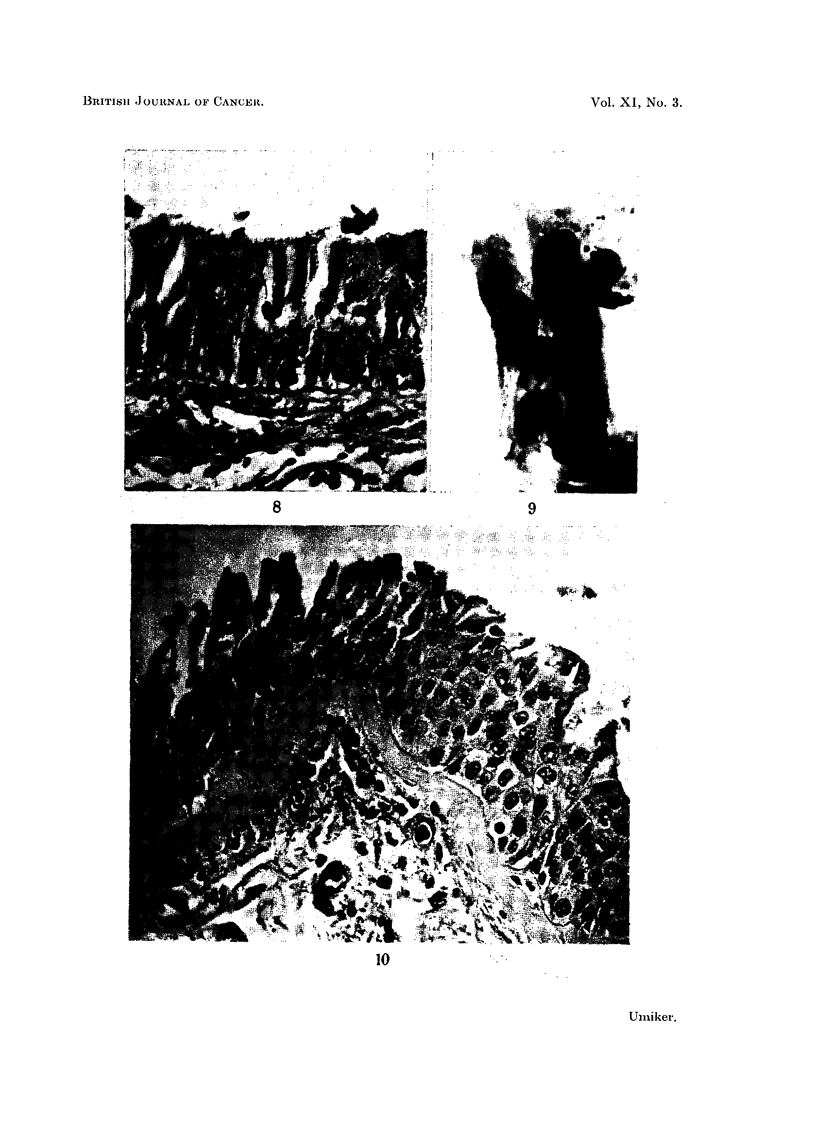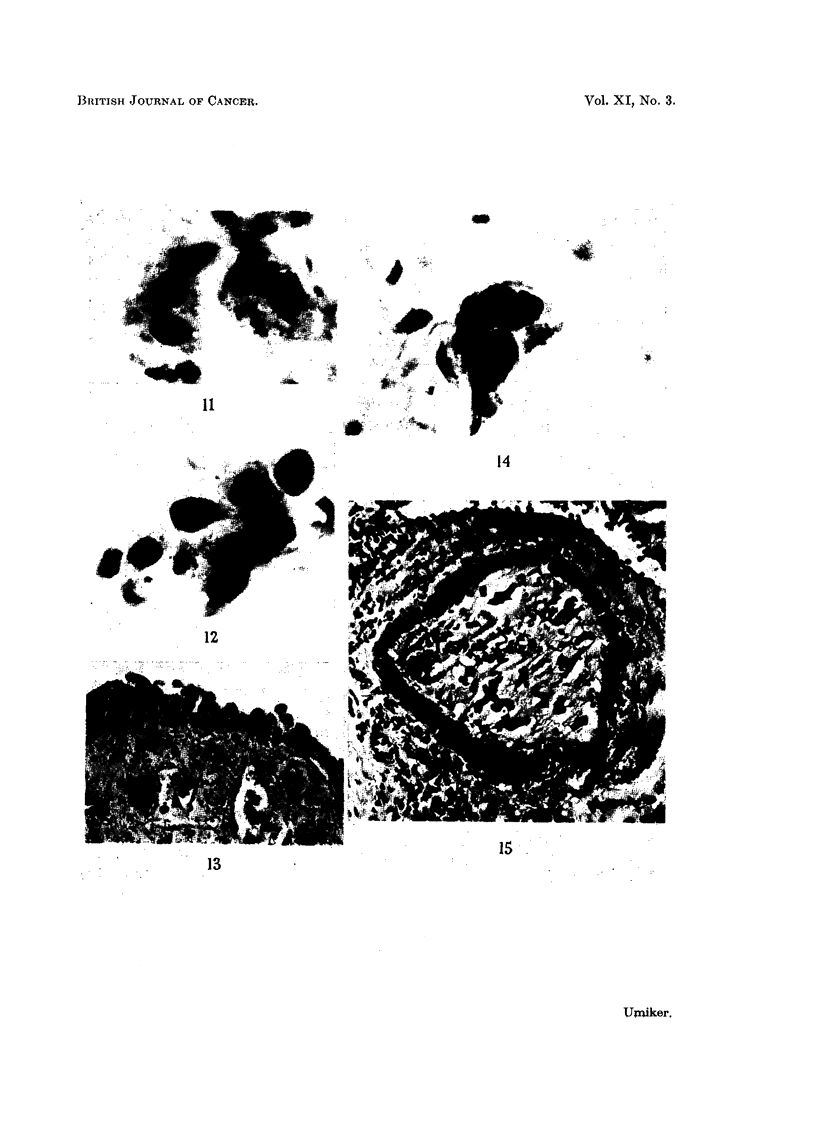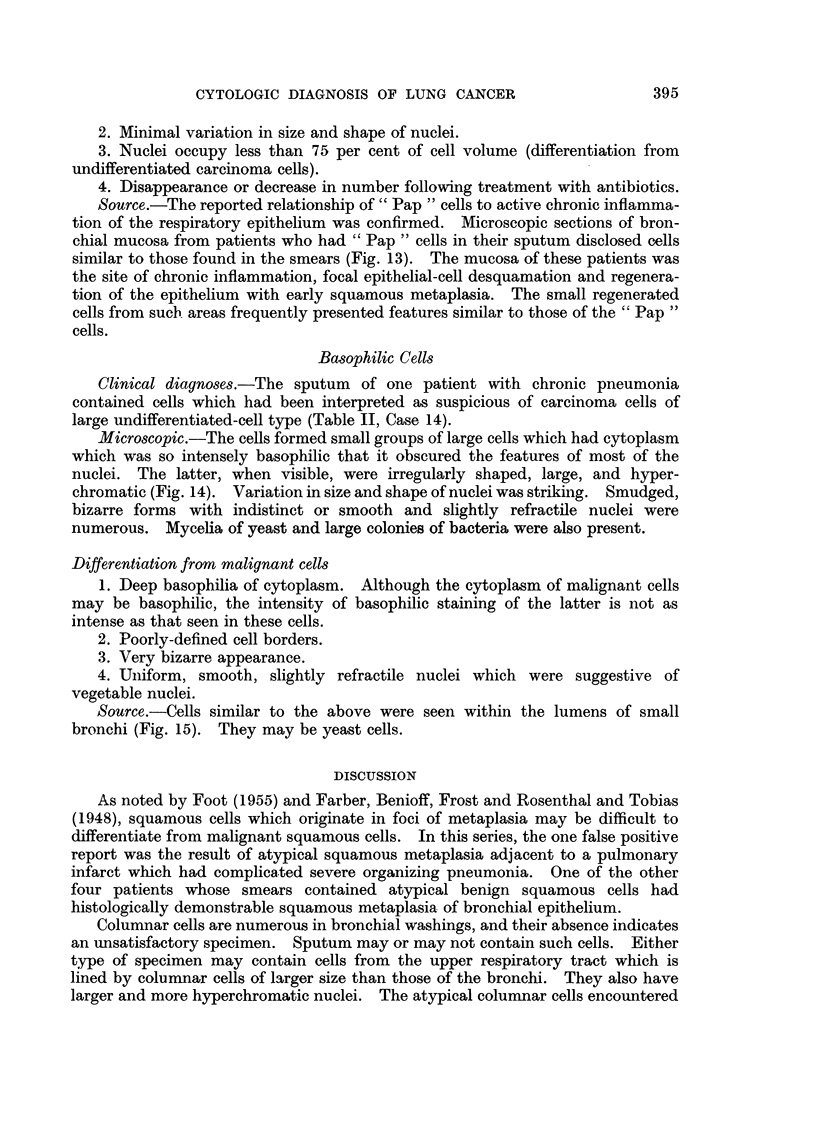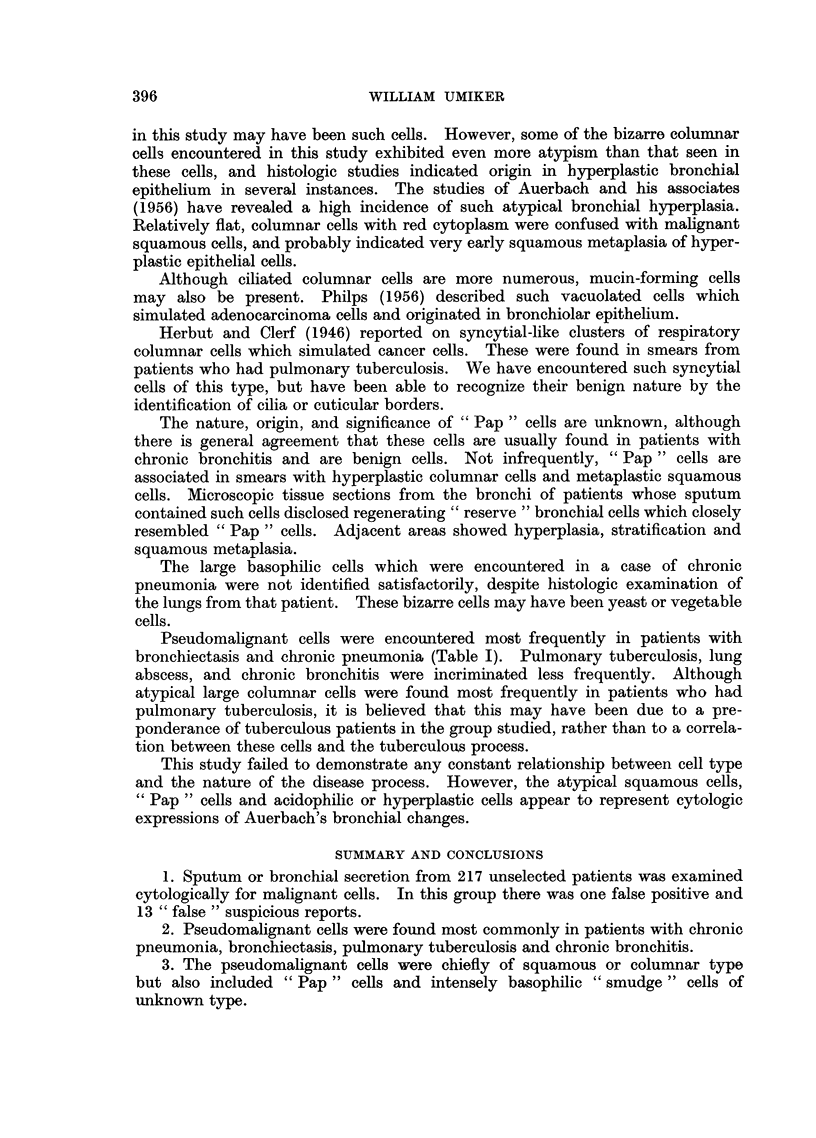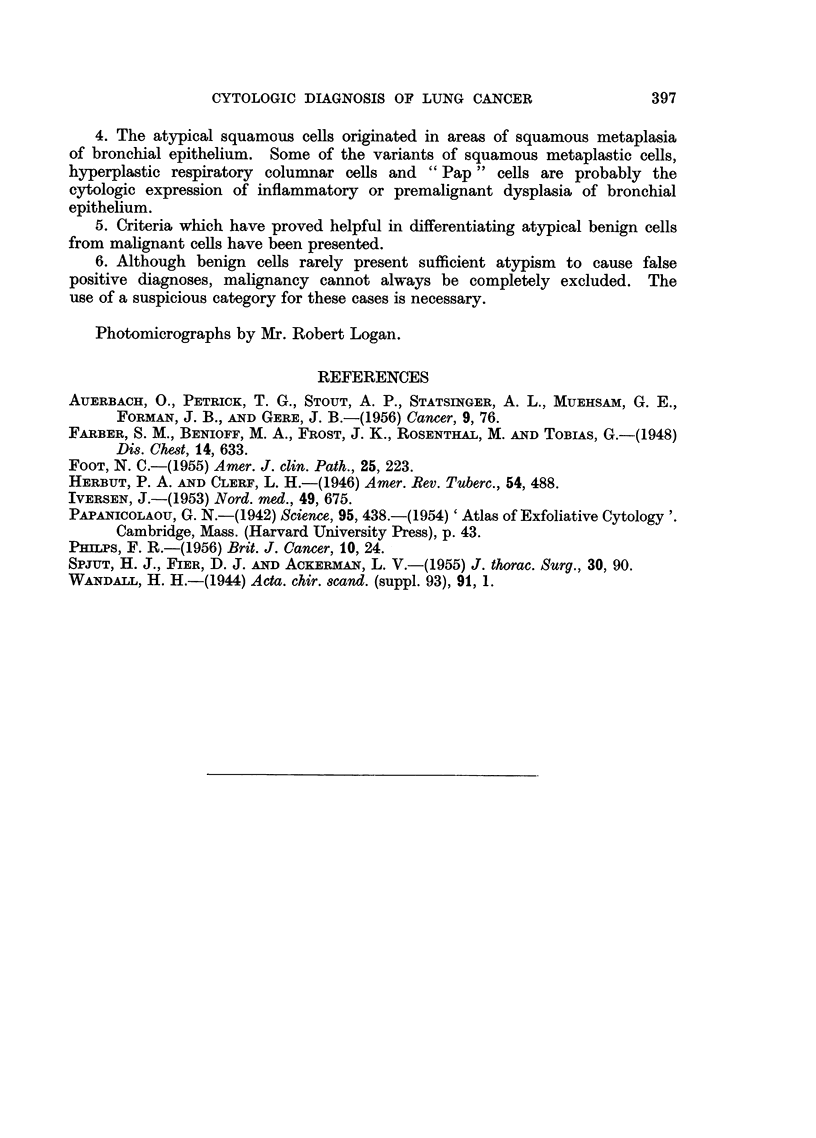# False Positive Reports in the Cytologic Diagnosis of Lung Cancer

**DOI:** 10.1038/bjc.1957.46

**Published:** 1957-09

**Authors:** William Umiker

## Abstract

**Images:**


### FALSE POSITIVE REPORTS IN THE CYTOLOGIC DIAGNOSIS OF LUNG CANCER

WILLIAM UMIKER

*Departments of Pathology, Veterans Administration Hospital and the University of Michigan Medical School, Ann Arbor, Michigan*

Received for publication June 18, 1957

THE value of exfoliative cytology in the diagnosis of lung cancer has been established, but the elimination of false positive reports has not been accomplished. In fact, it has been stated that a high degree of reliability can be achieved only through the acceptance of a lesser sensitivity (Farber, Benioff, Frost, Rosenthal and Tobias, 1948). Some reports lend support to this statement. For example, Spjut, Fier and Ackerman (1955) had no false positive smears of sputum or bronchial washings, but their original cytologic examinations were positive in only 243 of the 501 lung cancers. Conversely, Wandall (1944) detected cancer cells in the smears from all of his patients who had lung cancers from which he obtained adequate specimens, but he had false positive reports in almost 10 per cent of the cases. Nevertheless, according to Foot (1955), Papanicolaou and associates have achieved a sensitivity of over 95 per cent in the cytologic diagnosis of lung cancer despite a false positive rate of only 0·5 per cent.

Iversen (1953), who reviewed the literature and summarized the cytologic and clinical findings in 47 false positive cases, found false positive smears most frequently in patients with bronchiectasis, pneumonia or lung abscess (Table I). Foot (1955) also reported false positive smears due to bronchiectasis or pneumonia. Atypical squamous cells, large ciliated columnar cells, and " Pap " cells were responsible for most of his false positive results. Farber, Benioff, Frost, Rosenthal and Tobias (1948) reported that histiocytes may also simulate cancer cells.

This paper is a presentation of a study of benign atypical cells which simulate malignant cells in sputum or bronchial secretions.

TABLE I.—*Pulmonary Diseases Associated with False Positive or " False " Suspicious Cytologic Reports*

| | Iverson | Foot | Author | Total |
|---|---|---|---|---|
| Bronchiectasis | 8 | 5 | 2 | 12 |
| Pneumonia | 7 | 3 | 4 | 12 |
| Tuberculosis | 5 | 1 | 4 | 10 |
| Abscess | 8 | — | — | 8 |
| Bronchitis | 5 | — | 3 | 8 |
| Infarct | — | 1 | 1 | 2 |
| Others | 9 | 4 | — | 13 |

### MATERIAL AND METHOD

Smears of sputum or bronchial secretion from 217 patients, who were under investigation for possible lung cancer, had been examined previously for the presence of malignant cells. The spreads had been prepared according to the

Papanicolaou technique (1942). The smears from 53 patients had been reported as positive or suspicious for malignant cells. Thirty-nine of this group had proved bronchogenic cancer. The cytologic diagnosis of lung cancer could not be substantiated in the remaining 14 patients. The smears from one of these 14 patients had been interpreted as positive, and the other 13 suspicious for malignant cells. The smears from these 14 cases were reviewed and the atypical cells which had been incorrectly interpreted as positive or suspicious for malignant cells were studied.

### RESULTS

There were four types of cells which simulated cancer cells ; atypical squamous cells, " Pap " cells, large columnar cells and large basophilic " smudge " cells of undetermined nature.

### *Atypical Squamous Cells*

*Clinical diagnoses.*—Atypical squamous cells were encountered in five patients (Table II, Cases 1–5), including a patient (Case 1) whose false positive report resulted from chronic organizing pneumonia and pulmonary infarction with atypical squamous metaplasia of bronchioles (Fig. 1–4). One other patient in this group had a pulmonary infarct. The clinical diagnosis in two cases was chronic bronchitis. The fifth patient had severe bronchiectasis.

TABLE II.—*Pseudomalignant Cell Type and Clinical or Pathologic Diagnoses*

| Case | | |
|---|---|---|
| 1 | Squamous | Bronchiectasis. |
| 2 | ,, | Organizing pneumonia and infarct. |
| 3 | ,, | Chronic bronchitis. |
| 4 | ,, | ,, ,, |
| 5 | ,, | Infarct of lung. |
| 6 | Columnar | Bronchiectasis. |
| 7 | ,, | Chronic bronchitis. |
| 8 | ,, | Tuberculosis. |
| 9 | ,, | ,, |
| 10 | ,, | ,, |
| 11 | Columnar and " Pap " | Chronic pneumonia. |
| 12 | " Pap " | Tuberculosis. |
| 13 | ,, | Chronic pneumonia. |
| 14 | Large basophilic " smudge " cell | ,, ,, |

*Note.*—Case number 2 was reported as positive, all the others as suspicious for malignant cells.

*Microscopic.*—Atypical, benign, squamous cells were solitary (Fig. 1) or in clusters. They were usually polyhedral although oval, elliptical or " tadpole " forms were encountered. The abundant cytoplasm was intense red or orange and the cell borders were distinct. Occasionally there was disturbance of the nucleocytoplasmic ratio (Fig. 1). Although the nuclear chromatin was uniformly distributed in most cells, clumping of chromatin occasionally simulated that found in malignant cells (Fig. 1, 2, 5). Frequently, two or more overlapping nuclei simulated a solitary bizarre nucleus until the cell was examined more closely (Fig. 5). Nucleolar abnormalities were not marked.

*Differentiation from malignant cells*

1. Relative preservation of nucleocytoplasmic ratio : In most instances the cytoplasm was abundant. Small cells showed the greatest disturbance of nucleocytoplasmic ratio but their nuclei rarely occupied more than one-half of the cell volume.
2. Less variation in size and shape of cells and nuclei.
3. Regularity of nuclear borders : The borders of the nuclei of benign cells were usually uniform, smooth and regular, in contrast to the ragged, irregular borders of malignant cells. Fig. 1, 2 and 5 show unusual exceptions to this rule.
4. Less clumping of chromatin.
5. Absence of multiple features of malignancy in any one cell.

### *Columnar Cells*

*Clinical diagnoses.*—Columnar cells which resembled cells of adenocarcinoma resulted in four " false " suspicious results (Table II, Cases 6–9). The clinical diagnoses in these four cases were pulmonary tuberculosis (2), bronchiectasis and emphysema.

The smears from a patient with tuberculosis, and another set from a patient who had chronic pneumonia contained columnar cells which were interpreted originally as atypical squamous cells (Table I, Cases 10 and 11).

*Microscopic.*—Usually the cells were in clusters, palisades (Fig. 6) or, rarely, acini (Fig. 7). Cilia, a heavy cuticular border, pale translucent cytoplasm and tall columnar configuration characterize normal respiratory columnar cells. However, cells which were perpendicular to the slide appeared round or oval, the cytoplasm opaque and the cilia or cuticules invisible (Fig. 6). Others were devoid of cilia or cuticular borders and had cuboidal, polygonal or fusiform configurations. Multinucleation, large nuclei and large nucleoli were common findings. Atypical cells of this type simulated carcinoma cells of glandular type, especially when goblet cells were included in cell clusters (Fig. 6). Nuclear atypism was seldom striking, but it must be remembered that the cells of well-differentiated adenocarcinomas may not show marked pleomorphism.

In two cases the cytoplasm of flattened columnar cells was deeply acidophilic and the cells suggested atypical, keratinized, squamous cells (Fig. 9). However, their nuclei were similar to those of columnar cells and transition forms were identified. Histologic examination of the bronchial epithelium disclosed epithelial cell hyperplasia and changes suggesting very early squamous metaplasia (Fig. 10).

*Differentiation from malignant cells*

1. Presence of cilia or heavy cuticular membrane. It is debatable whether malignant respiratory cells ever have cilia but for practical purposes any cells with cilia should be regarded as a benign cell.
2. Preservation of polarity in the palisaded clusters.
3. Minimal nucleolar atypism.
4. Close association with normal ciliated columnar cells.
5. The presence of transition forms.

*Source.*—These cells resembled the large, columnar cells which line the upper respiratory passages, but in several instances origin in hyperplastic bronchial epithelium was possible (Fig. 8, 10).

### *" Pap " Cells*

Papanicolaou (1954) found small, elliptical, acidophilic cells with pyknotic nuclei in his own sputum. These cells have since been referred to as " Pap " cells and are found in patients with chronic bronchitis.

*Clinical diagnoses.*—Three patients in this series had " Pap " cells which resembled malignant cells of squamous or undifferentiated cell variety (Table II, Cases 11–13). The clinical diagnoses were chronic pneumonia (2) and pulmonary tuberculosis.

*Microscopic.*—These cells were found in small clumps of loosely joined cells or as isolated cells which measured less than 15 microns in diameter (Fig. 11, 12). Usually, the cytoplasmic borders were sharp and the nuclei small. Occasionally, the cells were blurred, smudged, or associated with " shadow " forms which appeared to be similar cells which had undergone degenerative or lytic change. The small size of these cells accentuated any nuclear enlargement, thereby producing sufficient nucleocytoplasmic disproportion to suggest malignancy, especially of the undifferentiated-cell type.

*Differentiation from malignant cells*

1. Absence of large bizarre squamous cells (differentiation from malignant squamous cells).

---

EXPLANATION OF PLATES

FIG. 1.—Atypical squamous cell with large nucleus, irregular nuclear border and prominent nucleolus. False positive smear from patient with pulmonary infarct. × 970.

FIG. 2.—Same case. Cluster of bizarre squamous cells exhibiting nuclear hyperchromasia, irregular nuclear borders, clumping of chromatin and variation in shape of nuclei. Note the abundant cytoplasm. × 970.

FIG. 3.—Same case. Histologic section showing chronic organizing pneumonitis and an infarct (top of photograph). Multiple islands and strands of metaplastic squamous cells. × 85.

FIG. 4.—Same case. Higher magnification of cells in Fig. 3. Marked atypism of some of the metaplastic squamous cells. × 970.

FIG. 5.—Atypical squamous cells. Overlapping nuclei in the upper cell are easily misinterpreted as a solitary nucleus of a malignant cell. False suspicious smear. Severe bronchiectasis. × 1080.

FIG. 6.—Overlapping columnar cells which resemble cells of adenocarcinoma. Note the goblet cell, with its excentric nucleus, in one corner of the cluster. False suspicious smear. Chronic pneumonia. × 1080.

FIG. 7.—Large cluster of columnar cells with pseudo-acinar arrangement. Note large nucleoli in a few cells. False suspicious smear. Emphysema. × 1080.

FIG. 8.—Microscopic section of bronchus showing epithelial hyperplasia with increased stratification and cells similar to those in Fig. 6 and 7. × 355.

FIG. 9.—Cluster of overlapping columnar cells which resemble squamous cells because of acidophilic cytoplasm, indistinct cilia and large nuclei. False suspicious smear. Pulmonary tuberculosis. × 1010.

FIG. 10.—Microscopic section of bronchus showing stratification, loss of cilia and early squamous metaplasia. The cytoplasm is acidophilic like that of the cells in Fig. 9. × 355.

FIG. 11.—Two " Pap " cells. Note that they are much smaller than the adjacent macrophage. Negative smear. × 1350.

FIG. 12.—Cluster of " Pap " cells which resemble the cells of undifferentiated carcinoma. Note the nucleocytoplasmic disproportion and indistinct cell borders. False suspicious smear. Pulmonary tuberculosis. × 920.

FIG. 13.—Same case. Microscopic section showing squamous metaplasia with atypical regenerating " reserve " bronchial cells which are probably " Pap " cells. × 325.

FIG. 14.—Cluster of large cells with deeply basophilic cytoplasm and smudged nuclei. False suspicious smear. Chronic pneumonia. × 730.

FIG. 15.—Same case. Microscopic section showing a small bronchus filled with similar cells and the mycelia of yeast. × 95.

1

2

3

4

Umiker.

5

6

7

Umiker.

8

9

10

Umiker.

11

14

12

15

13

Umiker.

2. Minimal variation in size and shape of nuclei.
3. Nuclei occupy less than 75 per cent of cell volume (differentiation from undifferentiated carcinoma cells).
4. Disappearance or decrease in number following treatment with antibiotics.

*Source.*—The reported relationship of " Pap " cells to active chronic inflammation of the respiratory epithelium was confirmed. Microscopic sections of bronchial mucosa from patients who had " Pap " cells in their sputum disclosed cells similar to those found in the smears (Fig. 13). The mucosa of these patients was the site of chronic inflammation, focal epithelial-cell desquamation and regeneration of the epithelium with early squamous metaplasia. The small regenerated cells from such areas frequently presented features similar to those of the " Pap " cells.

### *Basophilic Cells*

*Clinical diagnoses.*—The sputum of one patient with chronic pneumonia contained cells which had been interpreted as suspicious of carcinoma cells of large undifferentiated-cell type (Table II, Case 14).

*Microscopic.*—The cells formed small groups of large cells which had cytoplasm which was so intensely basophilic that it obscured the features of most of the nuclei. The latter, when visible, were irregularly shaped, large, and hyperchromatic (Fig. 14). Variation in size and shape of nuclei was striking. Smudged, bizarre forms with indistinct or smooth and slightly refractile nuclei were numerous. Mycelia of yeast and large colonies of bacteria were also present.

*Differentiation from malignant cells*

1. Deep basophilia of cytoplasm. Although the cytoplasm of malignant cells may be basophilic, the intensity of basophilic staining of the latter is not as intense as that seen in these cells.
2. Poorly-defined cell borders.
3. Very bizarre appearance.
4. Uniform, smooth, slightly refractile nuclei which were suggestive of vegetable nuclei.

*Source.*—Cells similar to the above were seen within the lumens of small bronchi (Fig. 15). They may be yeast cells.

### DISCUSSION

As noted by Foot (1955) and Farber, Benioff, Frost and Rosenthal and Tobias (1948), squamous cells which originate in foci of metaplasia may be difficult to differentiate from malignant squamous cells. In this series, the one false positive report was the result of atypical squamous metaplasia adjacent to a pulmonary infarct which had complicated severe organizing pneumonia. One of the other four patients whose smears contained atypical benign squamous cells had histologically demonstrable squamous metaplasia of bronchial epithelium.

Columnar cells are numerous in bronchial washings, and their absence indicates an unsatisfactory specimen. Sputum may or may not contain such cells. Either type of specimen may contain cells from the upper respiratory tract which is lined by columnar cells of larger size than those of the bronchi. They also have larger and more hyperchromatic nuclei. The atypical columnar cells encountered in this study may have been such cells. However, some of the bizarre columnar cells encountered in this study exhibited even more atypism than that seen in these cells, and histologic studies indicated origin in hyperplastic bronchial epithelium in several instances. The studies of Auerbach and his associates (1956) have revealed a high incidence of such atypical bronchial hyperplasia. Relatively flat, columnar cells with red cytoplasm were confused with malignant squamous cells, and probably indicated very early squamous metaplasia of hyperplastic epithelial cells.

Although ciliated columnar cells are more numerous, mucin-forming cells may also be present. Philps (1956) described such vacuolated cells which simulated adenocarcinoma cells and originated in bronchiolar epithelium.

Herbut and Clerf (1946) reported on syncytial-like clusters of respiratory columnar cells which simulated cancer cells. These were found in smears from patients who had pulmonary tuberculosis. We have encountered such syncytial cells of this type, but have been able to recognize their benign nature by the identification of cilia or cuticular borders.

The nature, origin, and significance of " Pap " cells are unknown, although there is general agreement that these cells are usually found in patients with chronic bronchitis and are benign cells. Not infrequently, " Pap " cells are associated in smears with hyperplastic columnar cells and metaplastic squamous cells. Microscopic tissue sections from the bronchi of patients whose sputum contained such cells disclosed regenerating " reserve " bronchial cells which closely resembled " Pap " cells. Adjacent areas showed hyperplasia, stratification and squamous metaplasia.

The large basophilic cells which were encountered in a case of chronic pneumonia were not identified satisfactorily, despite histologic examination of the lungs from that patient. These bizarre cells may have been yeast or vegetable cells.

Pseudomalignant cells were encountered most frequently in patients with bronchiectasis and chronic pneumonia (Table I). Pulmonary tuberculosis, lung abscess, and chronic bronchitis were incriminated less frequently. Although atypical large columnar cells were found most frequently in patients who had pulmonary tuberculosis, it is believed that this may have been due to a preponderance of tuberculous patients in the group studied, rather than to a correlation between these cells and the tuberculous process.

This study failed to demonstrate any constant relationship between cell type and the nature of the disease process. However, the atypical squamous cells, " Pap " cells and acidophilic or hyperplastic cells appear to represent cytologic expressions of Auerbach's bronchial changes.

### SUMMARY AND CONCLUSIONS

1. Sputum or bronchial secretion from 217 unselected patients was examined cytologically for malignant cells. In this group there was one false positive and 13 " false " suspicious reports.

2. Pseudomalignant cells were found most commonly in patients with chronic pneumonia, bronchiectasis, pulmonary tuberculosis and chronic bronchitis.

3. The pseudomalignant cells were chiefly of squamous or columnar type but also included " Pap " cells and intensely basophilic " smudge " cells of unknown type.

4. The atypical squamous cells originated in areas of squamous metaplasia of bronchial epithelium. Some of the variants of squamous metaplastic cells, hyperplastic respiratory columnar cells and "Pap" cells are probably the cytologic expression of inflammatory or premalignant dysplasia of bronchial epithelium.

5. Criteria which have proved helpful in differentiating atypical benign cells from malignant cells have been presented.

6. Although benign cells rarely present sufficient atypism to cause false positive diagnoses, malignancy cannot always be completely excluded. The use of a suspicious category for these cases is necessary.

Photomicrographs by Mr. Robert Logan.

### REFERENCES

AUERBACH, O., PETRICK, T. G., STOUT, A. P., STATSINGER, A. L., MUEHSAM, G. E., FORMAN, J. B., AND GERE, J. B.—(1956) *Cancer*, **9**, 76.
FARBER, S. M., BENIOFF, M. A., FROST, J. K., ROSENTHAL, M. AND TOBIAS, G.—(1948) *Dis. Chest*, **14**, 633.
FOOT, N. C.—(1955) *Amer. J. clin. Path.*, **25**, 223.
HERBUT, P. A. AND CLERF, L. H.—(1946) *Amer. Rev. Tuberc.*, **54**, 488.
IVERSEN, J.—(1953) *Nord. med.*, **49**, 675.
PAPANICOLAOU, G. N.—(1942) *Science*, **95**, 438.—(1954) 'Atlas of Exfoliative Cytology'. Cambridge, Mass. (Harvard University Press), p. 43.
PHILPS, F. R.—(1956) *Brit. J. Cancer*, **10**, 24.
SPJUT, H. J., FIER, D. J. AND ACKERMAN, L. V.—(1955) *J. thorac. Surg.*, **30**, 90.
WANDALL, H. H.—(1944) *Acta. chir. scand.* (suppl. 93), **91**, 1.